# **Environmental and population correlates of variation in short torpor use by wild hazel dormice (*Muscardinus avellanarius***)

**DOI:** 10.1007/s00442-025-05730-3

**Published:** 2025-06-03

**Authors:** Charlotte Armitage, Jonathan J. Bennie, Eleanor R. Scopes, Kristen Liptrot, Ian White, Nida Al-Fulaij, Robbie A. McDonald

**Affiliations:** 1 Environment and Sustainability Institute, University of Exeter, Penryn Campus, Penryn, TR10 9EZ UK; 2 Centre for Geography and Environmental Science, University of Exeter, Penryn Campus, Penryn, TR10 9EZ UK; 3 RWE Renewables UK Ltd, Beechwood Business Park, Inverness, IV2 3BW UK; 4 People’s Trust for Endangered Species, London, SW8 4BG UK

**Keywords:** Dormouse, Endothermy, Gliridae, Heterothermy, Hibernator, Hibernation, Torpor

## Abstract

Torpor is a life history strategy for conserving energy during unfavourable conditions. Hazel dormice *Muscardinus avellanarius* are known for prolonged winter hibernation but also employ short bouts of torpor of less than 24 hrs, independently of hibernation. In Great Britain, at the edge of the species’ range, there is marked spatial and temporal variation in short torpor among individuals and among populations, the causes and consequences of which are not well understood. We quantified variation in torpor use in the dormouse active season and identified individual and population-level correlates of torpor frequency. We analysed the data from the National Dormouse Monitoring Programme, comprising 53,953 observations of adult dormice in nest boxes on 381 sites over 25 years. Short torpor was more frequent at sites towards western Britain, at greater elevation, with more ancient woodland, and greater connectivity of broadleaf woodland. Dormice were more frequently torpid on colder and wetter days, and within colder and wetter seasons. Adult dormice observed in boxes containing young were rarely torpid, while adults alone were more frequently torpid. Lighter individuals were more frequently torpid earlier in the season (April–August) and heavier individuals later (September–October). Sites where short torpor was more frequent had lower counts of dormice and lower counts of breeding events. As a response to short and long-term variation in environmental conditions, short torpor is likely a mediator of environmental drivers of dormouse population dynamics. This is of particular relevance under climate change and at range edges, which already experience adverse environmental conditions.

**Supplementary Information:**

The online version contains supplementary material available at 10.1007/s00442-025-05730-3.

## Introduction

Endotherms maintain high and consistent body temperatures, although this comes at great thermoregulatory cost, particularly during periods when environmental conditions are unfavourable or resources are scarce. To mitigate these energetic costs, some mammals use temporal heterothermy (Ruf and Geiser 2015), of which there are several strategies (Nowack et al. 2023), with varying implications for life history (Wilsterman et al. 2021). Hibernation comprises multiday bouts of torpor with periodic arousals (Nowack et al. 2023). In temperate zones, hibernation is seasonal and occurs during periods of sustained cold and food shortage, when heat production is costlier (Vuarin and Yves 2014; Scopes et al. 2024). Some hibernators, however, also undergo torpor independently of the hibernation season, employing bouts of short torpor, usually with durations of less than 24 hours (Wilz and Heldmaier 2000; Geiser 2020; Nowack et al. 2023).

By reducing body temperature closer to ambient and lowering metabolic rate, individuals can save considerable energy during torpor use (Ruf & Geiser 2015; Geiser and Cooper 2023). In an analysis of torpor in 214 species (43 birds and 171 mammals), mean minimum body temperature varied by approximately 13°C (Ruf and Geiser 2015). Mean minimum torpor metabolic rate was approximately 35% of the basal metabolic rate (BMR) in daily heterotherms but only 6% of BMR in hibernators (Ruf and Geiser 2015). Torpor is not only an approach to energy saving, but can also be associated with avoidance of hazards, such as predators (Bieber and Ruf 2009), storms (Nowack et al. 2015) and fire (Nowack et al. 2016). Moreover, comparative analyses suggest that mammals utilising torpor are more resilient to extinction because of their reduced exposure to environmental stressors (Geiser and Turbill 2009; Liow et al. 2009; Hanna and Cardillo 2014).

Knowledge of the causes and consequences of torpor use might enable better understanding of the adaptive consequences of variation in strategies for temporal heterothermy. The drivers of torpor use in small mammals and birds have often been investigated experimentally in captive populations under controlled conditions, enabling careful examination of the interactions between temperature and food resource availability. With reduced food availability, captive African woodland dormice (*Graphiurus murinus)* reduced their activity levels at 25 °C and entered torpor at 10 °C (Webb and Skinner 1996). In captive populations of edible dormouse (*Glis glis*) and of spectacled dormouse (*Graphiurus ocularis),* both of which are winter hibernators, as well as the Djungarian hamster (*Phodus sungorus*), which by contrast is a daily heterotherm, lower ambient temperatures drove individuals into short and daily torpor, respectively (Ruf et al. 1991; Perrin and Ridgard 1999; Wilz and Heldmaier 2000). Information from the field on the determinants of torpor use for temperate species is somewhat limited (Doucette et al. 2012; Vuarin and Yves 2014), although direct and indirect field observations suggest that temperature and food availability are also correlates of torpor in wild populations of both birds and mammals in diverse biomes (Körtner and Geiser 2000; Smit and McKechnie 2010; Smit et al. 2011; Doucette et al. 2012).

The interacting costs and benefits of torpor use are manifest in multiple aspects of population ecology, as constrained energy budgets often require individuals to trade-off survival and energetically costly breeding. Species that employ torpor during their reproductive period are often found in highly seasonal, unpredictable environments with associated variation in food supplies (McAllan and Geiser 2014). As with other rodents, edible dormice invest heavily in reproduction when resources are abundant, such as during tree mast years, but in contrast to other rodents, when foraging opportunities are few they exhibit prolonged dormancy that extends to the active season (Bieber and Ruf 2009; Lebl et al. 2011). Edible dormice are prone to further, perhaps more subtle, trade-offs, and, for example, are unable to enter torpor because of testosterone production, but often sleep in groups; thereby, reducing energy expenditure through social thermoregulation (Fietz et al. 2004, 2010). Unusually, male edible dormice appear to present some constraint upon breeding success at a population level, as the number of reproductive males correlates with the number of litters produced (Fietz et al. 2004). In many rodent species, reproduction and torpor are mutually exclusive processes (McAllan and Geiser 2014). During hibernation, male edible dormice temporarily halt spermatogenesis, while torpor use in females delays foetal development (Fietz et al. 2004, 2010). For example, torpor is suppressed in male pouched mice (*Saccostomus campestris*) when they are producing testosterone, to facilitate opportunistic breeding but compromising energy conservation (Mzilikazi and Lovegrove 2002).

Some female small mammals use torpor during pregnancy, incubation and lactation (Johnson and Lacki 2014; McAllan and Geiser 2014). Torpor use during reproduction in females has been documented through observational studies in several small mammal species; Mulgaras (*Dasycercus blythi*) will enter torpor during gestation but not after (Körtner et al. 2008), whereas other species such as dunnarts (*Sminthopsis macroura*) and little brown bats (*Myotis lucifugus*) will enter torpor during all stages of reproduction including lactation (Geiser et al. 2005; Dzal and Brigham 2013). Although torpor expression when an individual is actively breeding results in an extended reproductive period, this is counter balanced by greater success in production of offspring and increased fitness of the parent (McAllan and Geiser 2014).

Hazel dormice *Muscardinus avellanarius* are small rodents, with a temperate, largely European range (Hutterer et al. 2021). They are primarily associated with broadleaf habitats, particularly early to mid-successional stages with diverse and dense structure, including scrub and traditionally managed woodlands, such as coppice (Juškaitis and Augutė 2008). They are hibernators, and undergo seasonal hibernation over the winter months, when conditions are cool and food is scarce, which, in Great Britain, is November to March. In addition to winter hibernation, they also employ short torpor during every month of the year when they are not hibernating, which, in Great Britain, is April to October (Bright and Morris 1996; Findlay-Robinson and Hill 2024). Female dormice in Britain usually have one litter later in the active season (after August) but, if conditions are favourable, they can also breed earlier in the season (between June and July) and can have two litters (Bright et al. 2006). Hazel dormice in Britain are at the north-western edge of the species’ distribution range, where climatic conditions are likely to be more energetically demanding (Pretzlaff and Dausmann 2012). Local weather conditions alter hazel dormice activity and in Britain individuals are thought to employ short torpor during cool, wet periods (Bright et al. 2006). In their continental range, dormice are more likely to be observed to be torpid on colder days, earlier in the year, and in individuals with lower body mass (Juškaitis 2005; Pretzlaff et al. 2014). Dormice are opportunistic feeders but cannot digest cellulose and so must exploit plants at different life stages throughout the season; they are therefore particularly sensitive to seasonal food shortages and are thought to employ short torpor to save energy during lean periods (Bright et al. 2006; Goodwin et al. 2020; Richards et al. 1984). Dormice therefore represent a useful model for investigating the causes and correlates of torpor during the active/reproductive season and the associated consequences for populations.

Hazel dormice are a species of conservation concern. They are a European Protected Species, listed under Annex IV of the European Habitats Directive (1992) and, in the UK, are afforded protection under the Habitats Regulations (1994). The species is categorised as Least Concern under the International Union for Conservation of Nature (IUCN) Red List and European Regional Assessment, however this assessment states that in parts of its northern range hazel dormice are declining and there is cause for concern (Hutterer et al. 2021). Dormouse populations appear to be in chronic decline in England and Wales, with an overall decline of 78% in counts of adults at nest box monitoring sites from 1994 to 2020 (Goodwin et al. 2017; Scopes et al. 2023). As a consequence, they are categorised as Vulnerable on the UK Red List for mammals (Mathews and Harrower 2020).

Although the overall trend in dormouse populations in Britain is one of decline (Goodwin et al. 2017; Scopes et al. 2023), local counts fluctuate between years, suggesting that dormice experience locally ‘good and bad years’. Hazel dormice have also been shown to be more abundant and to produce more litters on sites with warmer, sunnier springs (Goodwin et al. 2018). The connections between environmental conditions, use of short torpor, survival and breeding remain poorly understood. By analysing data from the National Dormouse Monitoring Programme (NDMP), which is an extensive, national, long-term dataset from summer nest box monitoring, our aim was to explore intrinsic and extrinsic, habitat and climate-related correlates of spatial and temporal variation in short torpor, with a view to improving understanding of the causes and consequences for hazel dormouse populations in Great Britain. We hypothesised that frequency of short torpor would be affected by environmental conditions, and would, in turn, relate to variation in dormouse counts and in breeding events.

## Methods

### National Dormouse Monitoring Programme

Hazel dormice are monitored in England and Wales with the National Dormouse Monitoring Programme (NDMP). The NDMP provides monitoring data that comprise a spatially and temporally extensive series of monthly counts, and snapshots of dormouse states and behaviours, in Great Britain. For a detailed description of the survey protocol, see PTES (2022) and for an analysis and validation of the monitoring programme, see Goodwin et al. (2017). In brief, permanent nest boxes are set up across sites, usually approximating a grid formation, depending on habitat and topography. Volunteers conduct nest box surveys predominantly in the morning (70% of recorded observations are 8:00–12:00), on monthly visits between April and October (mean visits per site per year=4.75, 95% CI 4.69–4.80), when dormice are active and not hibernating. Dormice very rarely use nestboxes for hibernation, as box temperatures are highly variable and ill-suited to hibernation; hence, the use of boxes is confined to the active season (Bright et al. 2006). Volunteers undergo extensive, closely-supervised training and typically complete two seasons of surveys to obtain the necessary survey license. Training enables them to distinguish between torpid and non-torpid dormice. Boxes are checked and dormice are sexed, weighed and their torpor state (torpid/not torpid) are recorded. Litters are also recorded. Individual dormice are not marked in this extensive monitoring scheme. The precise time of day of individual observations has not been recorded consistently and, despite this being a likely source of variation in observation of torpor (Juskaitis, 2005; Pretzlaff et al. 2014), for this study it remains part of residual, unexplained variation.

Only observations for adult dormice were used in this analysis as it has been shown that juveniles are less likely to enter torpor (Juškaitis 2005). Records were excluded from analysis if they omitted details of sex, weight and torpor state (torpid/not torpid), or contained erroneous data, for example negative weights. Records (n=1604) of hibernating dormice and of active dormice outside the active season (November–March) were removed. Records from 1993 to 2018 were included in the programme data, but data from 1988 to 1992 were excluded as the programme included relatively few sites (*n* <30) in its early years (Goodwin et al. 2017). Records from a site were removed from the dataset if fewer than 15 dormice were recorded over the whole study period. After this cleaning, of the original 77,048 observations of adult dormice, we used 53,953 from 381 sites in the analysis.

### Statistical analysis

All analysis was completed in R version 4.2.2 (R Core Team 2022).

### Correlates of torpor in dormouse populations

To investigate between-site, spatial variation in torpor use, we first created a site-level index of the frequency with which dormice were observed in short torpor, which controlled for variation arising from month, year and sex, and from variations in nest box sampling of the site, and of survey sampling of boxes. To derive this torpor index, we used a generalised linear model (GLM) with torpor as a binary response (torpid or not torpid) and a family link ‘logit’, and site, month, year and sex as predictor variables. To prevent zero inflation in the models, if no torpid individuals were recorded at a site over the study period, a single “dummy” torpid observation was added at the earliest survey date. This process ensured that no sites had unrealistically fitted probabilities of short torpor very close to zero, as it was considered that the true probability of short torpor occurring at any one site was non-zero. The coefficient for each site was extracted from the model (and inverse-logit transformed) and was used as the site torpor index, representing the likelihood of a dormouse being found in short torpor at a site, relative to an arbitrary reference site; hence, the higher the site torpor index, the greater the fitted likelihood of finding a dormouse in short torpor at that site on any given day or year. The reference site coefficient is, by default, zero, and therefore the (inverse-logit transformed) index is 0.5. Torpor index values above 0.5 represent a higher probability than the reference site, and vice versa. The reference site was NDMP site 450, which is located in Wiltshire (grid reference SU247689) and was chosen as this is the site closest to the mean latitude and longitude co-coordinate of all the sites in the dataset.

To investigate relationships between short torpor and environmental characteristics, GLMs were constructed using the site torpor index as a response, with a Gaussian error structure. Predictor variables were: latitude, longitude, elevation, solar index, proportion of ancient woodland, proportion of broadleaf, and woodland connectivity. The dredge function was used in R to obtain the most parsimonious combinations of these variables (Bartón, 2023). The importances of variables (the proportion of models within the top set within 2 AIC containing each variable) were compared to identify the variables likely to affect the relative probability of torpor at a site.

All NDMP sites have recorded point locations, though only a subset (*n*=264) have polygon data that identify the sampled area in which surveys take place. Only sites with this polygon information were included in the subsequent analysis. Location (latitude and longitude) for each site was obtained from the centroid of each polygon. To determine the proportion of broadleaf woodland at each site, the Forestry Commission’s National Forest Inventory (NFI) 2010–2020 was used (Forestry Commission 2022). For each of the NDMP sites, woodland area was calculated as the total area of connected woodlands not separated by >20 m of non-woodland habitat, taken as the mean over the period 2010–2020. This area may exceed the area of the sampled NDMP polygon, as it also includes adjacent woodland. An average proportion of broadleaf cover for the woodland areas was obtained from 2011 to 2020 and used as our variable. The proportion of each woodland area classified as ancient woodland was calculated as a separate variable (Spencer and Kirby 1992). These data were obtained from the Ancient Woodland Inventory, provided by Natural England and Natural Resources Wales (Welsh Government 2021; Natural England 2023). Connectivity was expressed as the total area of (i) broadleaf woodland and (ii) ancient woodland within a 1 km radius of the centroid of the site.

Topographical information was extracted from Terrain 50 digital terrain models from Ordnance Survey at a resolution of 50 m (Ordnance Survey 2022). From this we calculated the average elevation, aspect and slope for each site. The elevation data were centred (mean-subtracted) and scaled (divided by standard deviation) across sites. For each site and year, we calculated the ‘solar index’. This is a measure of the proportion of direct sunlight an area receives, using the R package ‘microclima’ (Maclean et al. 2019). For each site, the mean slope and aspect were obtained and the solar index calculated. A measure at 12pm on the first day of each month was calculated and then a mean was taken for each site per year. The solar index is calculated using the digital terrain model for a particular latitude, time of day, and day of the year, and varies between zero (the ground surface is in full shade) and one (the solar beam is perpendicular to the ground surface).

### Correlates of torpor in individual dormice

To investigate the extrinsic and intrinsic factors affecting torpor use in individual dormice, a binomial GLM was created. To explore the effects of local weather, we fitted a binomial GLM, using daily (short term), weekly (medium), monthly (long) and seasonal (90 days, very long) mean minimum temperatures (**°**C) and total rainfalls (mm) as explanatory variables.

We also analysed effects of dormouse characteristics. Body mass (g), sex (F, M), number of adults in the box, whether the adult dormouse was in a nest box with young dormice (breeding box) or no young (non-breeding box), the number of juveniles (young of the year independent of the mother) and number of dependent young in the box, were included as explanatory variables. The interactions between body mass and month, and between sex and month, were included, to test the effect of individual fitness and sex-specific variation in life-history, across the active season. We analysed variation in torpor use as a binary response with the family link ‘cloglog’ because of asymmetry in the distribution of torpid/not torpid (Thomas, 2015), since many more dormice were found to be active than in torpor.

For this analysis, slightly different data were used to those above; only observations with adult weights above 10g were included as any weights below this are likely to have been recording errors, and sites were excluded if only a single dormouse had been recorded across the duration of the scheme, as this was an individual level analysis. This resulted in 53,494 adult dormouse observations for analysis.

Minimum daily temperature and total daily rainfall for all NDMP sites for every survey year were obtained from the UK Met Office HadUK-Grid dataset, gridded at 1 x 1 km horizontal resolution. To investigate the short, medium, long and very long-term effects of local climate on dormice across a season, daily data climate variables were collated and then summarised for the 7, 30 and 90 days preceding and including the observation date, for each dormouse observation. This provided daily, weekly, monthly and seasonal average daily minimum temperature and total rainfall. These variables were chosen in accordance with climate variables found to be informative by Goodwin et al, (2018) when investigating variation in dormouse populations. A 30-year (1990–2020) baseline for central England was then subtracted from the climate data obtained from HadUK, resulting in site-level residuals from the England mean.

### Correlates of torpor and dormouse population characteristics

To investigate whether short torpor frequency might relate to variation in dormouse populations, we used site-level indices of dormouse abundance and of population trend, that controlled for survey effort (box numbers and visit frequencies), and accounted for between-year variation in counts, and derived these indices following the approaches applied by Goodwin et al., (2017) and updated by Scopes et al., (2023). We tested relationships between the site torpor index as a predictor and first the dormouse abundance index (log_10_ site-level index of dormice counted), and second an exponent of population trend index (site-level slope of population counts over time) as responses. For both analyses, a linear regression was built with Gaussian error structure.

To examine the potential impact of variation in torpor use on hazel dormouse populations, we derived a site torpor index similar to that above, but for each year of the survey, and for males and females separately (based on 27,292 observations of female and 29,286 male dormice). Treating the sexes separately, two binomial GLMs were built with site and year as an interaction term. The coefficients from the models were inverse logit, to scale yearly torpor scores resulting in a score between 0 (no torpor) and 1 for each site in each year.

Similar torpor indices were also created for early and late season. These were created in the same way as described above but only observations of dormice from April to July were included in the early season model and observations from August to October in the late season model. This resulted in four additional sets of scores; early and late season for males and for females.

We used eight measures as response variables to evaluate the effects of torpor use on hazel dormouse populations in both the current and subsequent seasons. We tested: total adult counts within a survey season; adult counts in the early and late season; counts of young (where these were the totals of all four age-classes of young); number of breeding events (defined as any instance where young dormice were found in a nest box); litter size; mean adult dormouse mass for each site and year (as a proxy for fitness) and mean mass of young. The mean young dormouse mass for each site was calculated using just the ‘greys-eyes-open’ age class, which are approximately 16–28 days old. We chose just this age class because it was the most commonly encountered, as at this age they are almost weaned from the mother. These measures were analysed using GLMs, with yearly torpor scores as predictor variables. Further details on the model structures are available in the supplementary information (Online Resource 1).

## Results

Of 53,953 observations, 23% were of dormice observed in short torpor. Monthly frequency of torpor varied widely, from 0.1 (September 2011, n*=*641 observations) to 100% (April 2010, *n*=30 observations).

### Correlates of torpor in dormouse populations

**Fig. 1.**
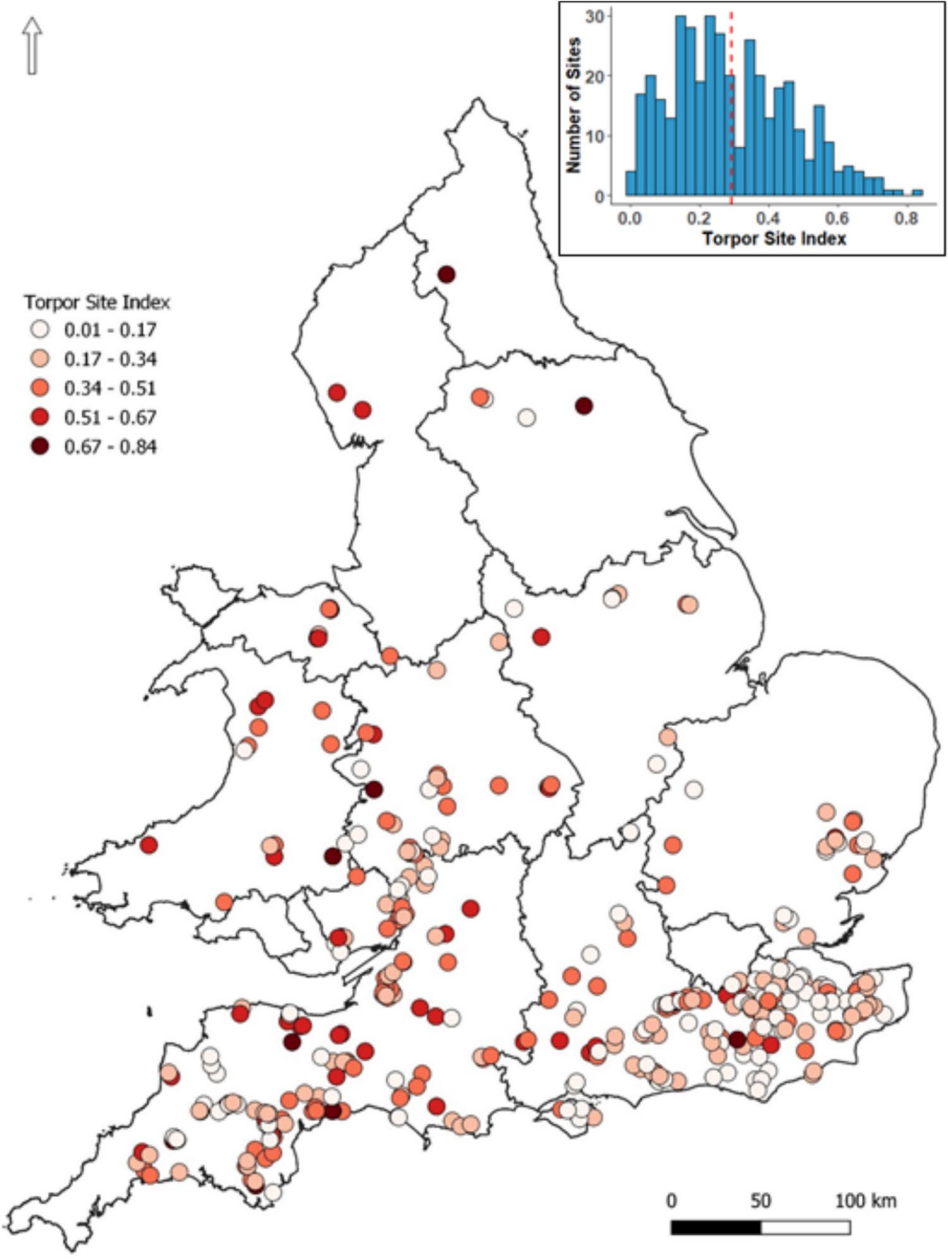
Locations of 381 National Dormouse Monitoring Programme sites in England and Wales and variation in the site-level index of short torpor frequency in hazel dormice. The site-level torpor index represents the likelihood of a dormouse being found in short torpor at a site, relative to an arbitrary reference site (site 450); hence, the higher index, the higher the fitted likelihood of finding a dormouse in torpor at that site on any given day or year. Observations are of dormice in nest boxes from the NDMP. Shade indicates the likelihood of finding a torpid dormouse at a site when compared with reference site 450. Darker sites represent more torpor at a site and lighter sites less torpor. Inset: Frequency histogram of torpor site index across all NDMP sites. Reference site 450 (central site), has a torpor site index of 0.5. The dotted red line denotes the mean site-level torpor index value across all NDMP sites that were analysed

**Fig. 2.**
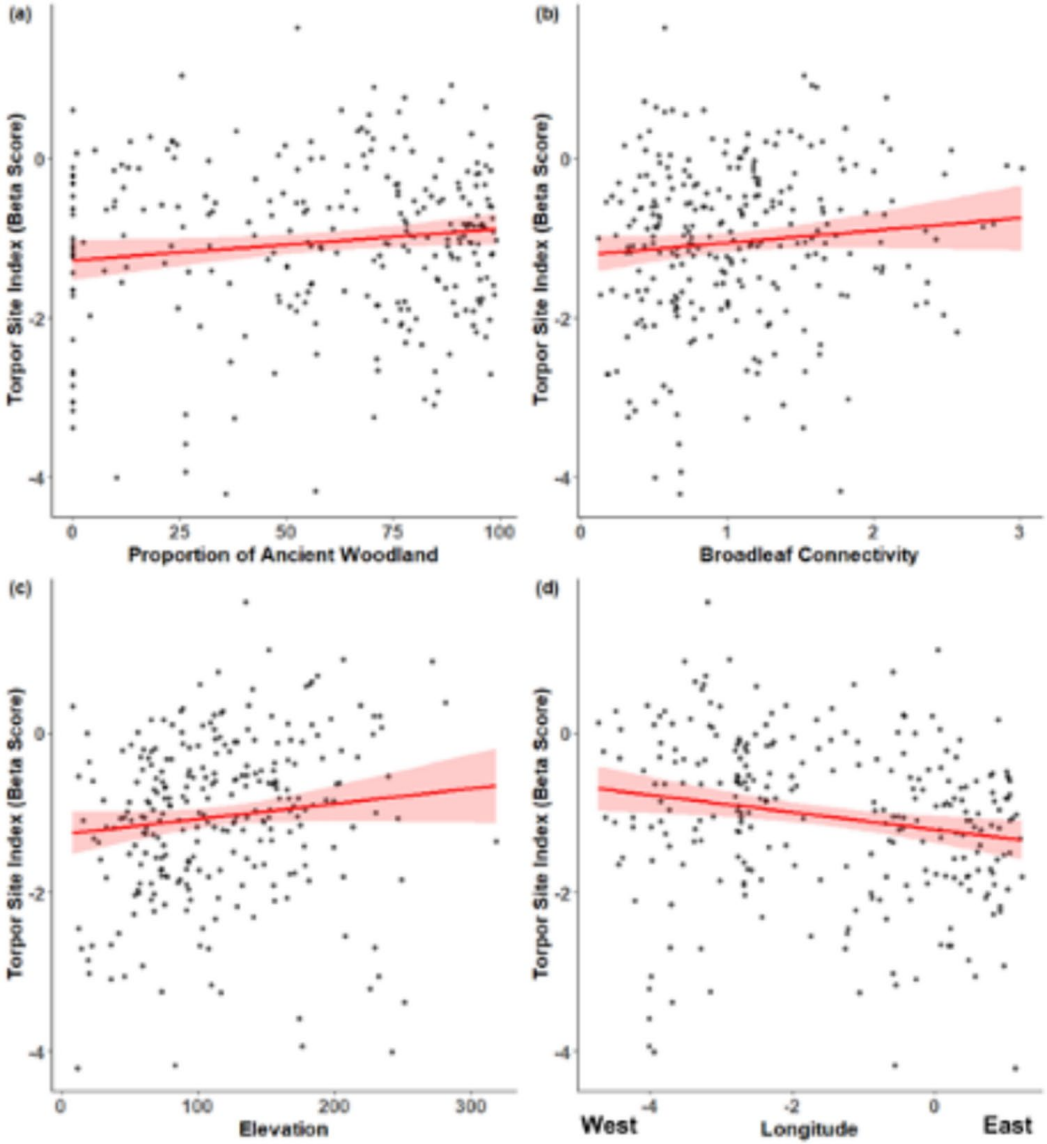
Relationships between the frequency of short torpor in hazel dormice and environmental conditions at sites from the National Dormouse Monitoring Programme. The torpor index is a site-level summary of the frequency of observations of hazel dormice in short torpor, controlling for survey effort and variation among years. Variables in the top performing model were: **a** proportion of ancient woodland, **b** connectivity of broadleaf woodland to a site, **c** elevation, and **d** longitude. Red lines denote the model prediction for each variable and bands illustrate the estimated standard error of the effect. Raw data are plotted in black, where each dot represents a single monitoring site

The torpor index was highly variable among NDMP sites (Figure 1). The best performing model of variation in the site torpor index based on site characteristics contained four variables; longitude, elevation, proportion of ancient woodland, and broadleaf connectivity. Ancient woodland connectivity, proportion of broadleaf, solar index, and latitude were not included in this model. The top model set contained 36 models and the four variables in the top model had the highest importance in this set (Table 1). Sites with a high torpor index were more likely to be in the west of Britain and at sites at higher elevations (Figure 2). Sites with a large proportion of ancient woodland and with higher connectivity of broadleaf habitat were also more likely to have a higher site torpor index (Figure 2).

**Table 1.** Summary of results of models analysing variation in the frequency of short torpor in hazel dormice among monitoring sites in England and Wales

Monitoring site characteristic	Importance in top model set	Weighted average of the co-efficient
Longitude (East–West location)	1	− 0.11
Proportion of ancient woodland	0.67	0.003
Elevation	0.67	0.001
Broadleaf connectivity	0.61	0.14
Latitude	0.53	0.06
Solar index	0.39	− 0.48
Ancient woodland connectivity	0.34	− 0.01
Proportion of broadleaf	0.08	0.0001

Summary is the top model set of environmental factors affecting a site-level index of short torpor. Observations are of dormice in nest-boxes from the National Dormouse Monitoring Programme. 36 models were in the top model set (within 2 AIC of the top performing model), the importance denotes the proportion of times each variable appeared in the top model set to indicate their relative influence over variation in the torpor index. The four variables that appeared most often in the top model set were also the four variables in the single top performing model

### Correlates of torpor in individual dormice

**Fig. 3.**
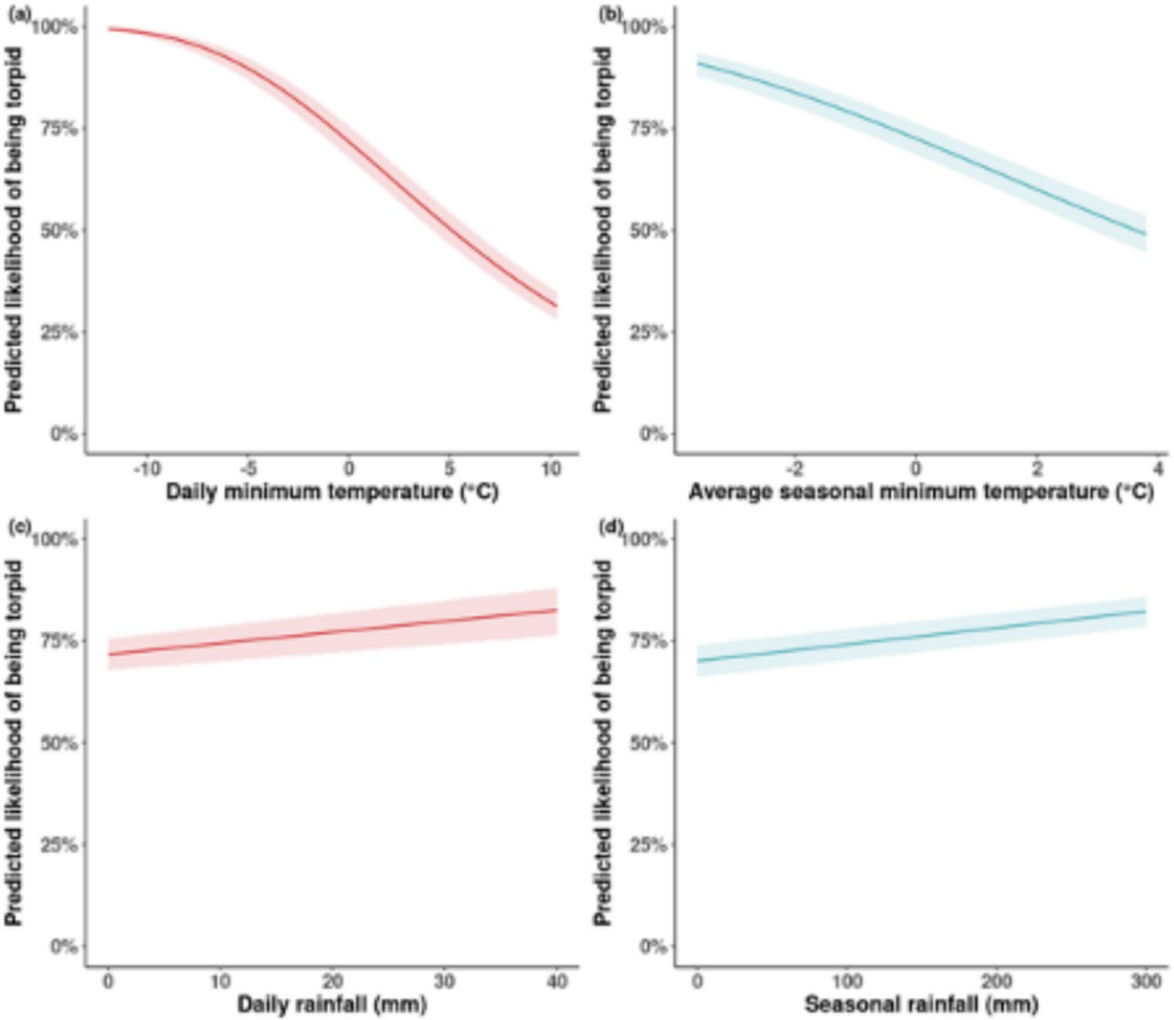
Relationships between daily and seasonal variation in environmental conditions on the likelihood of an adult hazel dormice being observed in short torpor. Plots indicate the predicted likelihood of finding a dormouse in torpor at different temporal scales for (**a**) daily and (**b**) seasonal minimum mean temperature and for (**b**) daily and (**c**) seasonal total rainfall, when all other variables are held constant. Predictions are from binomial GLM investigating the extrinsic and intrinsic factors affecting torpor in dormice. Observations were from the National Dormouse Monitoring Programme. All climatic variables are expressed as the difference from the central England 30-year baseline, so denote whether a site is cool/warm and dry/wet relative to the baseline. Shading around the line denotes the 95% confidence interval in the model predictions

When daily temperature is no different from a baseline of the mean and when all other variables are held constant, the likelihood of finding a dormouse torpid is 72% (95% CI: 68–75%) and if daily temperature is 4 °C warmer than baseline this decreases to 55% (95% CI: 51–59%) (Table 2, Figure 3). When seasonal temperature is at baseline, the likelihood of finding a dormouse torpid is 72% (95% CI: 69–76%) and if seasonal temperature is 4 °C warmer than baseline this decreases to 48% (95% CI: 43–52%) (Table 2; Figure 3). Weekly and monthly temperature variation did not significantly affect torpor use.

**Table 2.** Summary of results of model analysing variation in the tendency of hazel dormice to be found in short torpor, as predicted by extrinsic and intrinsic factors

	Coefficient	Standard error	Hazard ratio	*P*
April		Reference		
May	− 0.2102	0.2211	0.8104	0.3417
June	0.0393	0.2269	1.0401	0.8620
July	0.3654	0.3041	1.4410	0.2295
August	− 1.9246	0.4529	0.1459	0.0002
September	− 5.6565	0.4453	0.0035	<0.0001
October	− 4.9837	0.2215	0.0068	<0.0001
Weight (g)	− 0.0369	0.0113	0.9638	0.0011
Female		Reference		
Male	− 0.1851	0.0648	0.8310	0.0048
Box with no young		Reference		
Box with young	− 2.5216	0.1619	0.0803	<0.0001
Number of adults	− 0.1272	0.0120	0.8805	<0.0001
Number of juveniles	− 0.5805	0.0585	0.5596	<0.0001
May * Mass	− 0.0166	0.0130	0.9835	0.2015
June * Mass	− 0.0504	0.0134	0.9508	0.0002
July * Mass	− 0.1161	0.0177	0.8904	<0.0001
August * Mass	− 0.0611	0.0244	0.9407	0.0123
September * Mass	0.0973	0.0223	1.1021	<0.0001
October * Mass	0.1249	0.0121	1.1331	<0.0001
May * Males	0.0905	0.0724	1.0947	0.2113
June * Males	0.0841	0.0731	1.0877	0.2500
July * Males	0.1179	0.0890	1.1252	0.1849
August * Males	0.3920	0.1542	1.4800	0.0110
September * Males	− 0.4169	0.1748	0.6591	0.0171
October * Males	− 0.0205	0.0843	0.9797	0.8081
Daily minimum temperature	− 0.1175	0.0035	0.8891	<0.0001
Seasonal average minimum temperature	− 0.1715	0.0102	0.8424	<0.0001
Daily rainfall	0.0081	0.0020	1.0081	<0.0001
Season rainfall	0.0012	0.0001	1.0012	<0.0001

Model coefficients denote the effect size of predictor variables, standard error measures how precise the model estimates are, hazard ratio denotes the probability of torpor relative to the reference conditions, and P statistics denote the significance of each variable. Hazard ratio is included because of the cloglog error structure in the model. Hazard ratio denotes a measure that compares the likelihood of an event occurring in one group relative to another, with a value above 1 indicating higher risk and below 1 indicating lower risk in the first group

When daily rainfall is no different from a baseline of the mean and when all other variables are held constant, the likelihood of finding a dormouse torpid is 72% (95% CI: 68–76%) and if daily rainfall is 40 mm greater than baseline this increases to 83% (95% CI: 76–88%) (Table 2; Figure 3). When seasonal rainfall is at baseline, the likelihood of finding a dormouse torpid is 70% (95% CI: 66–74%) and if seasonal rainfall is 300mm greater than baseline this increases to 82% (95% CI: 79–86%) (Table 2, Figure 3). Weekly and monthly total rainfall variation did not significantly affect torpor.

**Fig. 4.**
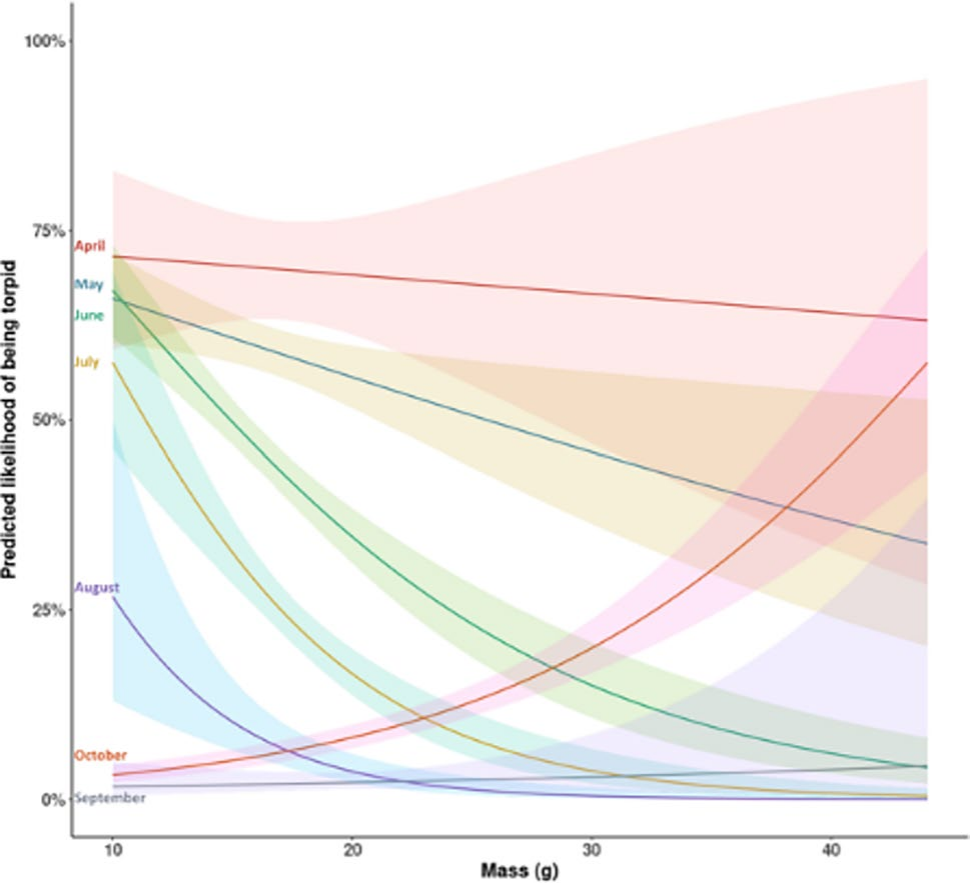
Relationships between body mass and month and the likelihood of an adult hazel dormouse being observed in short torpor. The plot indicates the predicted likelihood of finding a dormouse in torpor during different months of the active season (April–October), based on an individual’s mass (g), when all other variables are held constant. Predictions are from binomial GLM investigating the extrinsic and intrinsic factors affecting torpor in dormice. Observations were from the National Dormouse Monitoring Programme. Shading around the line denotes the 95% confidence interval in the model predictions

The relationship between mass and the likelihood of being in torpor varied by month; from April to August lighter individuals were more likely to be in torpor, whereas in September and October this pattern reversed and heavier individuals were more likely to be in torpor (Table 2, Figure 4). The relationship between sex and the prevalence of torpor use varied by month; males were more likely than females to be in torpor in August, though this effect reversed in September, when females were more likely torpid. Across other months, April–July and October, there was no significant difference between the sexes in their use of torpor.

Adult dormice found in nest boxes with young dormice were significantly less likely to be in torpor than those in boxes with no young, with a 91% reduction (95% CI: 89–94%) in the incidence of torpor (Table 2). As the number of adults or juveniles found in the box increased, then the probability of torpor use decreased (Table 2). The number of young found in the box did not significantly affect torpor use.

An ROC curve using a threshold of 0.5 was plotted to validate the model of environmental correlates of torpor use, and the resulting area under the curve was 86%. The ROC curve shows how well a binary classification model distinguishes between two classes by visualising the trade-off between the true positive rate and the false positive rate. The area under the ROC curve (AUC) provides a single metric summarising this performance; an AUC of 100% represents a perfect model, therefore our model was performing well.

### Correlates of torpor and dormouse population characteristics

#### Abundance and population trend

**Fig. 5.**
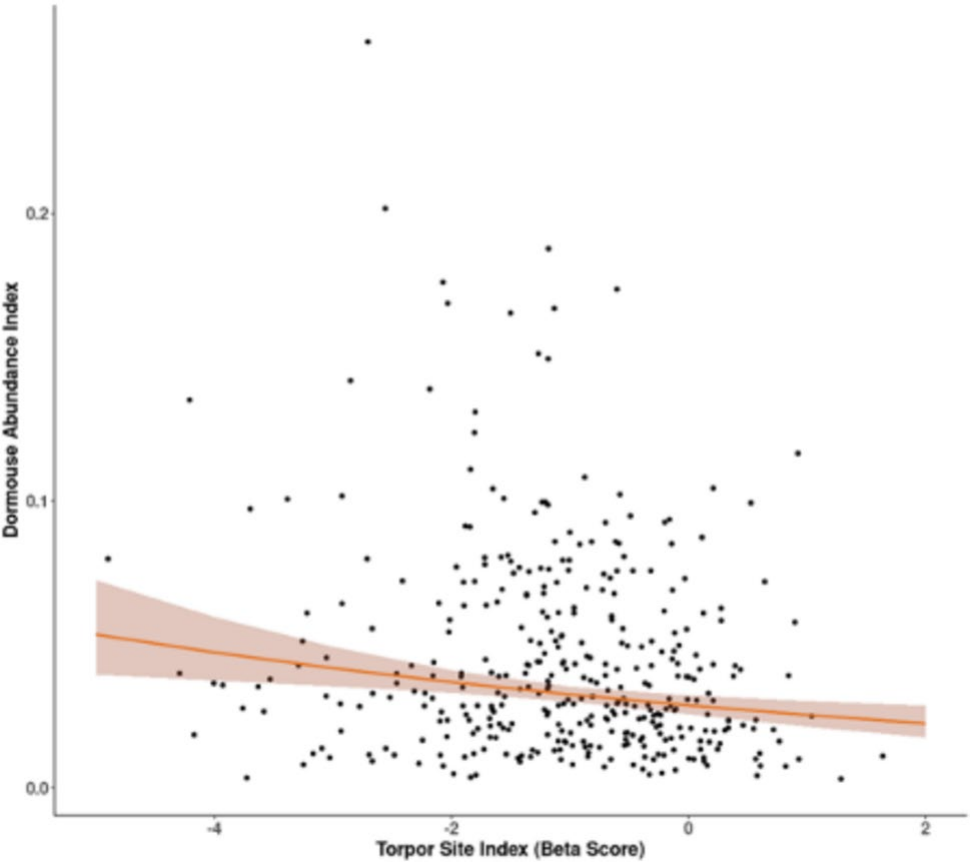
Relationship between the frequency of short torpor in hazel dormice and dormouse counts at sites from the National Dormouse Monitoring Programme. The torpor index is a site-level summary of the frequency of observations of dormice in short torpor, and the abundance index is a site-level summary of adult dormouse counts, both controlling for survey effort and variation among years. Observations were from the National Dormouse Monitoring Programme. The relationship between indices of torpor and abundance is shown as the orange line and the abundance index has been back transformed. Confidence intervals are denoted by lighter orange shading and raw data are black points. The higher the torpor index the greater the likelihood of dormice being observed in torpor

There was a significant, negative relationship between the site-level indices of dormouse torpor use and dormouse abundance (*P*=0.0014; Figure 5), but not with population trend.

**Fig. 6.**
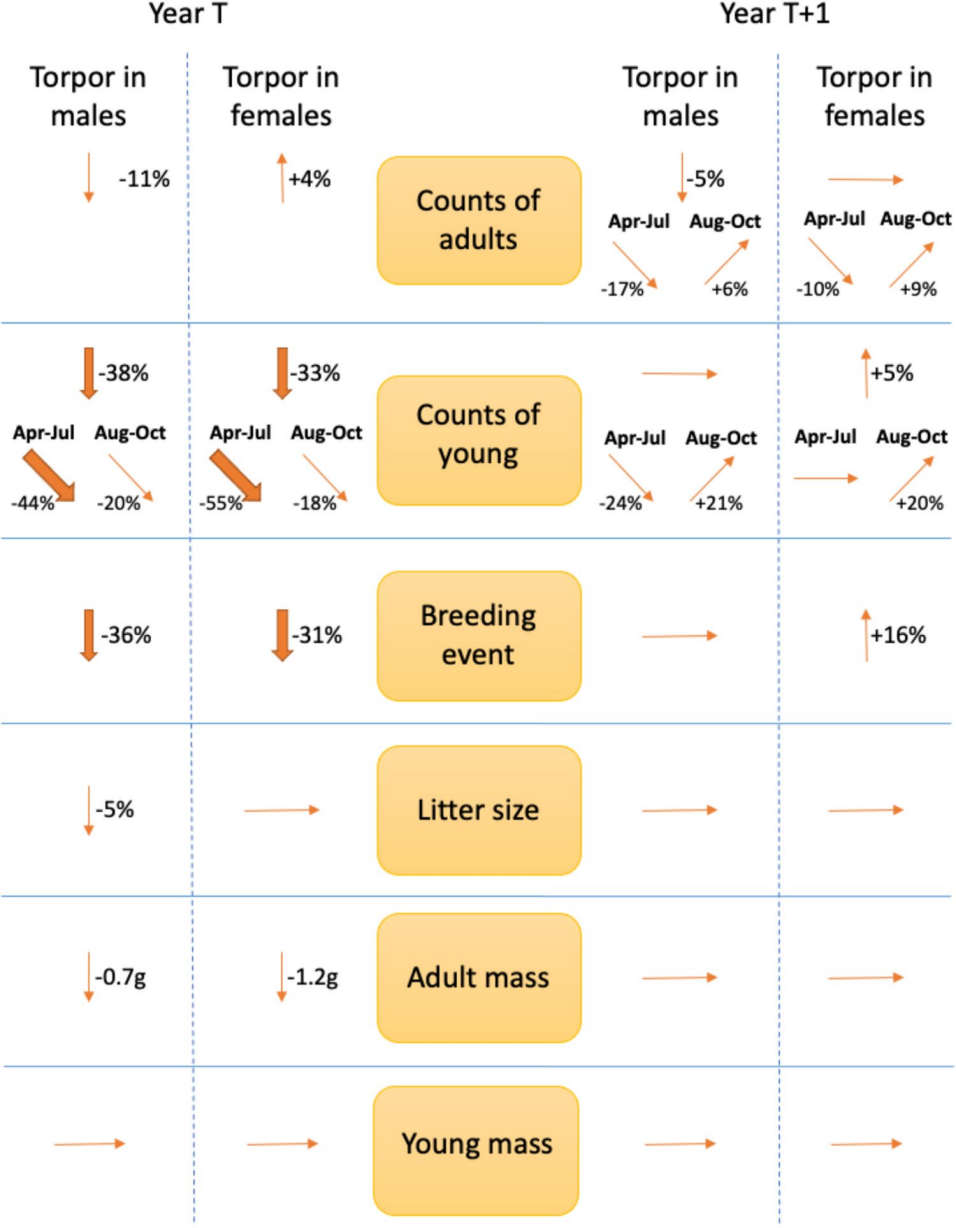
Summary of relationships between short torpor frequency and population parameters in hazel dormice. Observations were from the National Dormouse Monitoring Programme. Treating the sexes separately, two binomial GLMs were built with site and year as an interaction term. Central boxes denote the population parameter that was tested as a response variable. Columns on the left summarise relationships between high frequency of torpor in either male or female dormice and the corresponding parameter. Columns on the right summarise relationships between torpor in year T and the population parameter in the year T+1. An upward arrow signifies a positive relationship, an orange downward arrow a negative relationship, and a horizontal arrow indicates no significant relationship. Counts of adults and counts of young correspond to the whole survey year, and to when split between early and late parts of the season. Percentages are comparisons of the differences in the population parameter between sites where torpor was very rare (torpor index of 0) and those where it was very frequent (torpor index of 1)

The magnitudes of effects are described by comparing predicted outcomes when there is a torpor score of 0 (very infrequent torpor) and a torpor score of 1 (very frequent torpor) between the sexes (Figure 6).

#### Adult counts

Relationships between torpor frequency and adult dormouse counts differed between the sexes. Frequent torpor use in males was associated with an 11% (95% CI: 8–13%) decrease in adult counts in the same active season, while frequent torpor use in females was associated with a 4% (95% CI: 1–6%) increase. Frequent torpor use in males was associated with a 5% (95% CI: 3–7%) decrease in counts of adults in the subsequent year, although female torpor use had no similar effect. The relationship between torpor frequency and adult counts in the subsequent year varied over the course of the active season. High rates of torpor use in males and females resulted in a reduction in counts of adults early in the season in the subsequent year of 17% (95% CI: 14–21%) and 10% (95% CI: 6–13%), respectively. In contrast, high rates of torpor use in males and females resulted in an increase in counts of adults late in the season in the subsequent year of 6% (95% CI: 2–10%) and 9% (95% CI: 6–13%), respectively.

#### Breeding and litter size

Frequent torpor use in males and females was associated in the same year with reductions in counts of young of 38% (95% CI: 35–40%) and 33% (95% CI: 30–35%), respectively. Frequent torpor use in females resulted in a 5% (95% CI: 1–9%) increase in counts of young in the subsequent year, though male torpor use had no similar effect. Frequent torpor use in males in the early season was associated with a 44% (95% CI: 38–49%) reduction in counts of young, while frequent torpor use in females in the early season was associated with a 55% (95% CI: 50–61%) reduction. Frequent torpor use in males and females in the late season was associated with reductions in counts of young of 20% (95% CI: 17–23%) and 18% (95% CI: 14–21%), respectively.

The relationship between torpor frequency and counts of young varied over the course of the active season in the following year. High rates of torpor use among males resulted in a reduction in counts of young early in the season, in the following year, by 24% (95%CI: 18–35%), female torpor use had no similar effect. In contrast, later in the season, high rates of torpor use in males and females resulted in similar increases in counts of young the following year (21% 95% CI: 11–32% and 20% 95% CI: 12–28%, respectively).

Frequent torpor use in males and females was associated with reductions in the number of breeding events in-year of 36% (95% CI: 30–41%) and 31% (95% CI: 25–36%), respectively. Frequent torpor use in females was associated with an increase in the number of breeding events in the subsequent year of 16% (95% CI: 7–25%), though male torpor had no similar effect.

Frequent torpor use in males was associated with a reduction in litter size in-year of 5% (95%CI: 0%-9%), though female torpor had no similar effect. Torpor frequencies for both sexes had no effect on litter sizes in the subsequent year.

#### Body mass

Frequent torpor use was associated with lower mean adult masses in-year in both males (− 0.7 g, 95% CI: 0.4–1.0 g) and females (− 1.2 g, 95% CI: 0.9–1.5 g). Torpor frequency had no effect on adult masses in the subsequent year. Torpor use had no effect on the masses of young in either the same year or the following year, irrespective of sex.

## Discussion

With this large scale and long-term study, we found that, in addition to the winter hibernation for which they are notorious, hazel dormice use short torpor in a highly variable manner. This variation relates to potential environmental drivers, including site location, elevation and habitat, and both short-term and seasonal variation in temperature and rainfall. Dormouse characteristics, including sex and their association with other adult and young dormice, also contribute. A complex pattern of interactions characterises the relationships between variation in short torpor use, breeding parameters and adult population counts. This does not necessarily suggest that torpor use is a direct driver of population variation. Rather, population characteristics are likely driven by environmental fluctuations, but via individual variation in their use of short torpor, alongside that in winter hibernation, and the trade-offs these both require with survival and breeding.

We show that cold, wet conditions, at local and regional spatial scales, and at daily and seasonal (but not weekly or monthly) temporal scales, were associated with more frequent use of short torpor. Moreover, we show that sites and years experiencing such conditions, and sites where dormice exhibit more frequent short torpor, were characterised by reduced counts of adults and reduced evidence of breeding. Sites where dormice were more frequently in torpor therefore appear to indicate climatic and habitat conditions that are relatively unfavourable for dormice and thus sustain smaller populations.

Cooler temperatures and higher rainfall correlated with greater frequency of short torpor use, signifying that dormice respond quickly to short-term conditions to save energy. Hazel dormice have a small body size and high conductance, and so are prone to losing more body heat in cool and humid conditions, hence they reduce their nightly activity when there is high rainfall (Bright et al., 1996). Instead of foraging in these costly conditions, dormice may mitigate energy losses by utilising torpor. While daily temperature and rainfall was related to torpor use, conditions during the week and month preceding the observation date had no such effect, but those over a whole season did. Thus dormice respond to both proximate conditions, by using short torpor to avoid bad weather, and to long-term conditions, likely in consequence of either their body condition determined over a period of months or more generalised impacts on plant phenology, productivity and food availability.

Environmental conditions and their energetic consequences, and how these relate to variation in torpor use at the level of individuals and populations, have the potential to add to understanding of species ranges. Species at the edge of their range exist in areas that are often associated with declining suitability and abundance of habitat (Caissy et al., 2020) and have a higher severity and frequency of extreme climatic events when compared with the core (Rehm et al., 2015). The edge of the range is therefore often more energetically demanding and unfavourable conditions prevent further expansion in these challenging environments. Employing heterothermy may enable endotherms to persist in areas that would otherwise be energetically unfavourable (Dausmann and Warnecke 2016). Opportunistic torpor use might even aid in the colonisation of new areas as energy requirements are reduced during challenging conditions and thereby enable a population to become established (Nowack and Dausmann 2015). This is particularly important under a changing climate as areas in a landscape might become more or less energetically favourable. In the case of hazel dormice in Britain, if environmental conditions become more favourable at what are currently the cooler and wetter margins, a reduction in the frequency of short torpor could be associated with growth of dormouse populations, where appropriate habitat is available (Juškaitis and Augutė 2008).

Associations between short torpor frequency and counts of adult hazel dormice are suggestive either of general relationships between frequent short torpor and demographic parameters (reduced survival within or between years), or extended hibernation (in the earliest and latest parts of the active season), or of potential variation in patterns of nest box usage. Notably, perhaps, such effects appear to vary with sex, reducing the likelihood of the latter sampling bias. Frequent short torpor in males was linked to lower adult counts within the same season, whereas frequent torpor in females was linked to increased adult counts, within the same season. Thus, male and female dormice appear to use short torpor differently, with a likelihood of distinct trade-offs between conditions, survival and breeding. Differing physiological and behavioural patterns with regards to torpor have been documented in other species. Male and female kalutas (*Dasykaluta rosamondae*) differ markedly in their use of short torpor in winter, likely associated with complete male die-off following mating; while females showed prolonged overnight bouts of torpor, males entered short and shallow bouts, and expended more energy while active (Körtner et al. 2010).

The dynamic patterns of trade-offs faced by males and females were particularly apparent later in the season. Relative to females, male dormice were more likely to be observed in short torpor in August but less likely in September (Juškaitis 2005; Pretzlaff et al. 2014), suggesting that reproductively active females might not routinely utilize short torpor. Our results further show relationships between torpor frequency and productivity as fewer young are found in years with more frequent torpor use. Somewhat counterintuitively, however, litter size is only affected by male torpor frequency and not that of females, perhaps because dormice exhibit multiple paternity in their litters (Naim et al., 2011). Other studies have suggested that there is a relationship between potential male partners and litter sizes in mammal species that have multiple paternity, specifically, when fewer male partners are available, females tend to have smaller litters (Dobson et al., 2018; Gayet et al., 2016). Increased torpor use by female dormice was associated with an increase in breeding the following year and an increase in adult counts later in that same year. This pattern may result from females making up for lost reproductive opportunities in the past year, or from unrelated density dependent effects (Combe et al. 2023).

The prevalence of short torpor in hazel dormice is influenced by the number of individuals found in nest boxes during monitoring. Solitary animals were more likely to exhibit torpor compared to those found in multiple occupancy boxes. Little is known about the social structure and behaviour of hazel dormice so it is difficult to surmise why this might be (Glass 2017). One possibility is that huddling enables behavioural thermoregulation, or that lowering metabolic rate and body temperature when entering torpor is more easily done when solitary (Fietz et al. 2010).

Hazel dormice are opportunistic feeders and their diets are highly variable driven by variation in food availability (Juškaitis 2007; Goodwin et al. 2020). In periods of scarcity, it would be expected that individuals sustain reduced body mass and hence are more likely to use short torpor. The relationship between mass and torpor use changes throughout the season. Lighter individuals enter torpor from April to August though this pattern shifts in September and October when heavier individuals were more likely to be found in torpor, as animals approach conditions suitable for hibernation (Juškaitis 2005). Dormice in Great Britain do not hibernate in nest boxes but go to ground where climatic conditions are more stable (Gubert et al. 2023; Findlay-Robinson and Hill 2024), therefore dormice found torpid in boxes in September and October are still exhibiting short torpor. Torpor frequency later in the dormouse active season therefore appears to be a positive indicator of local conditions as it indicates that individuals have built up enough reserves to enter hibernation and survive the winter. This is further supported by our findings that adults in sites with high torpor indices are lighter, suggesting that they are not entering winter hibernation in best condition. A study of garden dormice (*Eliomys quercinus*) in a captive setting demonstrated that late-born juveniles that were intermittently fasted used torpor to grow and accumulate fat prior to hibernation (Giroud et al. 2014). These possibilities all lend themselves to experimentation, or more detailed and individual-based studies across diverse sites.

Our results give an indication of the types of site that exhibit high or low levels of torpor use with regards to habitat. Dormice living on sites with more ancient woodland were more often in torpor. Perhaps counterintuitively, this could be because species composition or management practices in ancient woodland present fewer foraging opportunities to hazel dormice. Sites with greater connectivity of broadleaf habitats also had greater torpor site index scores, it remains unclear why this would influence dormancy as dormice are thought to prefer high levels of habitat connectivity for foraging and dispersal (Bright et al. 2006). Future research should focus on investigating how foraging opportunities vary across different habitat types and whether these variations influence the differences in torpor use observed in dormouse populations at different sites.

To understand the dynamics of torpor use and the significance of adverse or changing conditions, higher resolution information about torpor duration, and about other factors, including consequences of sampling (e.g. time of day), would be useful in giving a fuller account of variation in torpor use. This would allow for more powerful analysis into the environmental drivers of torpor use. When conditions are challenging for dormice, as with other species, there is an apparent trade-off between survival and investment in productivity (Bright and Morris 1996), and as a relatively long lived species (for a small rodent), fitness might be increased by postponing breeding to subsequent years. Our study highlights the links between the proximate trade-offs for daily energy budgets, and how these might eventually relate to wider population dynamics. Several studies have investigated the proximate relationships between daily torpor and breeding (McAllan and Geiser 2014). To our knowledge these mechanistic studies have not then related how these energy saving life–history manifestations affect population dynamics at scale. Increasing our understanding of these relationships is of particular importance as many species are facing a changing climate. As climate changes, for dormice and other users of short torpor, so will the frequency of their use of torpor, with potentially profound effects on species life history and populations.

## Supplementary Information

Below is the link to the electronic supplementary material.

Supplementary file1 (DOCX 27 KB)

## Data Availability

The data underpinning these analyses will be made available upon request to People's Trust for Endangered Species.

## Code availability

The source code used to produce the results presented in this paper is available on Zenodo data repository at (will add at acceptance).

## References

[CR1] Bartoń K 2023 MuMIn: multi-model inference. R package version 1.47.5. Available at: https://CRAN.R-project.org/package=MuMIn

[CR2] Bieber C, Ruf T (2009) Summer dormancy in edible dormice *(Glis glis)* without energetic constraints. Naturwissenschaften 96(1):165–171. 10.1007/s00114-008-0471-z 19034404 10.1007/s00114-008-0471-z

[CR3] Bright P, Morris PA (1996) Why are dormice rare? A case study in conservation biology. Mammal Rev 26(4):157–187. 10.1111/j.1365-2907.1996.tb00151.x

[CR4] Bright P, Morris PA, Mitchell-Jones T (2006) The dormouse conservation handbook, 2nd edn. Joint Nature Conservation Committee, Peterborough

[CR64] Caissy P, Klemet-N’Guessan S, Jackiw R, Eckert CG, Hargreaves AL (2020) High conservation priority of range-edge plant populations not matched by habitat protection or research effort. Biol Conserv 249:108732. 10.1016/j.biocon.2020.108732

[CR5] Combe FJ et al (2023) Density and climate effects on age-specific survival and population growth: consequences for hibernating mammals. Anim Conserv 26(3):317–330. 10.1111/acv.12843

[CR7] Dausmann KH, Warnecke L (2016) Primate torpor expression: ghost of the climatic past. Physiology 31(6):398–408. 10.1152/physiol.00050.2015 27708046 10.1152/physiol.00050.2015

[CR66] Dobson FS, Abebe A, Correia HE, Kasumo C, Zinner B (2018) Multiple paternity and number of offspring in mammals. Proc Royal Soc B: Biol Sci 285(1891):20182042. 10.1098/rspb.2018.2042 10.1098/rspb.2018.2042 PMC6253375 30429308

[CR8] Doucette LI, Brigham RM, Pavey CR, Geiser F (2012) Prey availability affects daily torpor by free-ranging Australian owlet-nightjars *(Aegotheles cristatus)*. Oecologia 169(2):361–372. 10.1007/s00442-011-2214-7 22173484 10.1007/s00442-011-2214-7

[CR9] Dzal YA, Brigham RM (2013) The tradeoff between torpor use and reproduction in little brown bats *(Myotis lucifugus)*. J Comp Physiol B 183(2):279–288. 10.1007/s00360-012-0705-4 22972361 10.1007/s00360-012-0705-4

[CR11] Fietz J, Schlund W, Dausmann KH, Regelmann M, Heldmaier G (2004) Energetic constraints on sexual activity in the male edible dormouse *(Glis glis)*. Oecologia 138(2):202–209. 10.1007/s00442-003-1423-0 14608499 10.1007/s00442-003-1423-0

[CR12] Fietz J, Klose SM, Kalko EKV (2010) Behavioural and physiological consequences of male reproductive trade-offs in edible dormice *(Glis glis)*. Naturwissenschaften 97(10):883–890. 10.1007/s00114-010-0704-9 20697882 10.1007/s00114-010-0704-9

[CR13] Findlay-Robinson R, Hill DL (2024) Hibernation nest site selection but not overwinter activity is associated with microclimatic conditions in a hibernating mammal. J Thermal Biol 123:103909. 10.1016/j.jtherbio.2024.103909 10.1016/j.jtherbio.2024.103909 39084175

[CR6] Forestry Commission. 2022. *National forest inventory NFI 2010-2020.* Forestry Commission. Available at: https://data-forestry.opendata.arcgis.com/. Accessed 1 Apr 2022

[CR65] Gayet T, Devillard S, Gamelon M, Brandt S, Say L, Baubet E (2016) On the evolutionary consequences of increasing litter size with multiple paternity in wild boar (*Sus scrofa scrofa*). Ecol 70(6): 1386–1397. 10.1111/evo.12949 10.1111/evo.12949 27166953

[CR14] Geiser F (2020) Seasonal expression of avian and mammalian daily torpor and hibernation: not a simple summer-winter affair. Front Physiol 11:436. 10.3389/fphys.2020.00436 32508673 10.3389/fphys.2020.00436 PMC7251182

[CR15] Geiser F, Cooper CE (2023) Daily torpor, hibernation, and heterothermy in marsupials. In: Cáceres NC, Dickman CR (eds) American and Australasian marsupials. Springer International Publishing, Cham, pp 1221–1248

[CR16] Geiser F, Turbill C (2009) Hibernation and daily torpor minimize mammalian extinctions. Naturwissenschaften 96(10):1235–1240. 10.1007/s00114-009-0583-0 19578825 10.1007/s00114-009-0583-0

[CR17] Geiser F, McAllan BM, Brigham RM (2005) Daily torpor in a pregnant dunnart (*Sminthopsis macroura* Dasyuridae: Marsupialia). Mamm Biol 70(2):117–121. 10.1016/j.mambio.2004.06.003

[CR18] Giroud S, Zahn S, Criscuolo F, Chery I, Blanc S, Turbill C, Ruf T (2014) Late-born intermittently fasted juvenile garden dormice use torpor to grow and fatten prior to hibernation: consequences for ageing processes. Proceedings of the Royal Society B: Biological Sciences 281(1797):20141131. 10.1098/rspb.2014.1131 10.1098/rspb.2014.1131 PMC4240977 25377448

[CR19] Glass D (2017) The social structure of the hazel dormouse *(Muscardinus avellanarius)*. University of Brighton, Brighton, UK

[CR20] Goodwin CED, Hodgson DJ, Al-Fulaij N, Bailey S, Langton S, McDonald RA (2017) Voluntary recording scheme reveals ongoing decline in the United Kingdom hazel dormouse *Muscardinus avellanarius* population. Mammal Rev 47(3):183–197. 10.1111/mam.12091

[CR21] Goodwin CED et al (2018) Climate, landscape, habitat, and woodland management associations with hazel dormouse *Muscardinus avellanarius* population status. Mammal Rev 48(3):209–223. 10.1111/mam.12125

[CR22] Goodwin CED, Swan GJF, Hodgson DJ, Bailey S, Chanin P, McDonald RA (2020) Effects of food availability on the trophic niche of the hazel dormouse *Muscardinus avellanarius*. For Ecol Manage 470–471:118–215. 10.1016/j.foreco.2020.118215

[CR23] Gubert L et al (2023) Using high-resolution LiDAR-derived canopy structure and topography to characterise hibernaculum locations of the hazel dormouse. Oecologia 202(4):641–653. 10.1007/s00442-023-05429-3 37543993 10.1007/s00442-023-05429-3 PMC10474991

[CR24] Hanna E, Cardillo M (2014) Clarifying the relationship between torpor and anthropogenic extinction risk in mammals. J Zool 293(3):211–217. 10.1111/jzo.12136

[CR25] Hutterer R, Kryštufek B, Yigit N, Mitsainas G, Meinig H, Juškaitis R 2021 *Muscardinus avellanarius* (amended version of 2016 assessment): The IUCN red list of threatened species 2021. Available at: https://www.iucnredlist.org/species/13992/197519168. Accessed 15 Jul 2023

[CR26] Johnson JS, Lacki MJ (2014) Effects of reproductive condition, roost microclimate, and weather patterns on summer torpor use by a vespertilionid bat. Ecol Evol 4(2):157–166. 10.1002/ece3.913 24558571 10.1002/ece3.913 PMC3925379

[CR27] Juškaitis R (2005) Daily torpor in free-ranging common dormice *(Muscardinus avellanarius)* in Lithuania. Mamm Biol 70(4):242–249. 10.1016/j.mambio.2005.02.007

[CR28] Juškaitis R (2007) Feeding by the common dormouse *(Muscardinus avellanarius)*: a review. Acta Zoologica Lituanica 17(2):151–159. 10.1080/13921657.2007.10512827

[CR29] Juškaitis R, Augutė V (2008) Habitat requirements of the common dormouse *(Muscardinus avellanarius)* and the fat dormouse *(Glis glis)* in mature mixed forest in Lithuania. Ekológia (Bratislava) 27(2):143–151

[CR30] Körtner G, Geiser F (2000) Torpor and activity patterns in free-ranging sugar gliders *Petaurus breviceps * (Marsupialia). Oecologia 123(3):350–357. 10.1007/s004420051021 28308589 10.1007/s004420051021

[CR31] Körtner G, Pavey CR, Geiser F (2008) Thermal biology, torpor, and activity in free-living Mulgaras in arid zone Australia during the winter reproductive season. Physiol Biochem Zool 81(4):442–451. 10.1086/589545 18507533 10.1086/589545

[CR32] Körtner G, Rojas AD, Geiser F (2010) Thermal biology, torpor use and activity patterns of a small diurnal marsupial from a tropical desert: sexual differences. J Comp Physiol B 180(6):869–876. 10.1007/s00360-010-0459-9 20217093 10.1007/s00360-010-0459-9

[CR33] Lebl K, Rotter B, Kürbisch K, Bieber C, Ruf T (2011) Local environmental factors affect reproductive investment in female edible dormice. J Mammal 92(5):926–933. 10.1644/10-MAMM-A-225.1

[CR34] Liow LH, Fortelius M, Lintulaakso K, Mannila H, Stenseth NC (2009) Lower extinction risk in sleep-or-hide mammals. Am Naturalist 173(2):264–272. 10.1086/595756 20374142 10.1086/595756

[CR35] Maclean IMD, Mosedale JR, Bennie JJ (2019) Microclima: an r package for modelling meso- and microclimate. Methods Ecol Evol 10(2):280–290. 10.1111/2041-210X.13093

[CR36] Mathews F, Harrower C 2020 IUCN—compliant Red List for Britain’s terrestrial mammals. Assessment by the Mammal Society under contract to Natural England, Natural Resources Wales and Scottish Natural Heritage. Natural England, Peterborough. Available at: https://www.mammal.org.uk/science-research/red-list/. Accessed 4 May 2022

[CR37] McAllan BM, Geiser F (2014) Torpor during reproduction in mammals and birds: dealing with an energetic conundrum. Integr Comp Biol 54(3):516–532. 10.1093/icb/icu093 24973362 10.1093/icb/icu093

[CR38] Mzilikazi N, Lovegrove B (2002) Reproductive activity influences thermoregulation and torpor in pouched mice, *Saccostomus campestris*. J Comp Physiol B 172(1):7–16. 10.1007/s003600100221 11824404 10.1007/s003600100221

[CR67] Naim DM, Telfer S, Sanderson S, Kemp SJ, Watts PC (2011) Prevalence of multiple mating by female common dormice, *Muscardinus avellanarius*. Conserv Genet 12(4):971–979. 10.1007/s10592-011-0200-6

[CR10] Natural England. 2023. Ancient Woodland (England). Available at: https://environment.data.gov.uk/arcgis/rest/services/NE/AncientWoodlandEngland/FeatureServer.

[CR39] Nowack J, Dausmann KH (2015) Can heterothermy facilitate the colonization of new habitats? Mammal Rev 45(2):117–127. 10.1111/mam.12037

[CR40] Nowack J, Rojas AD, Körtner G, Geiser F (2015) Snoozing through the storm: torpor use during a natural disaster. Sci Rep 5(1):11243. 10.1038/srep11243 26073747 10.1038/srep11243 PMC4466894

[CR41] Nowack J, Cooper CE, Geiser F (2016) Cool echidnas survive the fire. Proc Royal Soc B: Biol Sci 283(1828):20160382. 10.1098/rspb.2016.0382 10.1098/rspb.2016.0382 PMC4843662 27075255

[CR42] Nowack J, Stawski C, Geiser F, Levesque DL (2023) Rare and opportunistic use of torpor in mammals—an echo from the past? Integr Comp Biol 63(5):1049–1059. 10.1093/icb/icad067 37328423 10.1093/icb/icad067 PMC10714912

[CR56] Ordnance Survey 2022 OS Terrain 50. Ordnance survey. Available at: https://beta.ordnancesurvey.co.uk/products/os-terrain-50. Accessed 1 Jun 2022

[CR43] Perrin MR, Ridgard BW (1999) Thermoregulation and patterns of torpor in the spectacled dormouse,* Graphiurus ocularis* (A. Smith 1829) (Gliridae). Tropical Zool 12(2):253–266. 10.1080/03946975.1999.10539392

[CR44] Pretzlaff I, Dausmann KH (2012) Impact of climatic variation on the hibernation physiology of *Muscardinus avellanarius*. In: Ruf T, Bieber C, Arnold W, Millesi E (eds) Living in a seasonal world. Springer, Berlin, Heidelberg, pp 85–97

[CR45] Pretzlaff I, Rau D, Dausmann HK (2014) Energy expenditure increases during the active season in the small, free-living hibernator *Muscardinus avellanarius*. Mammalian Biol 79(3):208–214. 10.1016/j.mambio.2013.12.002

[CR46] PTES. 2022. National dormouse monitoring programme (NDMP)

[CR47] R Core Team 2022 R: A language and environment for statistical computing. Available at: https://www.R-project.org/

[CR63] Rehm EM, Olivas P, Stroud J, Feeley KJ (2015) Losing your edge: climate change and the conservation value of range‐edge populations. Ecol Evol 5(19):4315–4326. 10.1002/ece3.1645 26664681 10.1002/ece3.1645 PMC4667833

[CR48] Richards CGJ, White AC, Hurrell E, Price FEF (1984) The food of the common dormouse, *Muscardinus avellanarius*, in South Devon. Mammal Rev 14(1):19–28. 10.1111/j.1365-2907.1984.tb00335.x

[CR49] Ruf T, Geiser F (2015) Daily torpor and hibernation in birds and mammals. Biol Rev 90(3):891–926. 10.1111/brv.12137 25123049 10.1111/brv.12137 PMC4351926

[CR50] Ruf T, Klingenspor M, Preis H, Heldmaier G (1991) Daily torpor in the Djungarian hamster *(Phodopus sungorus)*: interactions with food intake, activity, and social behaviour. J Comp Physiol B 160(6):609–615. 10.1007/BF00571257

[CR51] Scopes ER et al (2023) Shifting baselines for species in chronic decline and assessment of conservation status. Are hazel dormice *Muscardinus avellanarius* Endangered? Ecol Solut Evid 4(1):e12206. 10.1002/2688-8319.12206

[CR52] Scopes ER, Broome A, Walsh K, Bennie JJ, McDonald RA (2024) Conservation implications of hibernation in mammals. Mammal Rev. 10.1111/mam.12346

[CR53] Smit B, McKechnie AE (2010) Do owls use torpor? Winter thermoregulation in free-ranging pearl-spotted owlets and African scops-owls. Physiol Biochem Zool 83(1):149–156. 10.1086/605457 19929636 10.1086/605457

[CR54] Smit B, Boyles JG, Brigham RM, McKechnie AE (2011) Torpor in dark times: patterns of heterothermy are associated with the lunar cycle in a nocturnal bird. J Biol Rhythms 26(3):241–248. 10.1177/0748730411402632 21628551 10.1177/0748730411402632

[CR55] Spencer JW, Kirby KJ (1992) An inventory of ancient woodland for England and Wales. Biol Conserv 62(2):77–93. 10.1016/0006-3207(92)90929-H

[CR62] Thomas R (2015) Data analysis with R statistical software: A guidebook for scientists. Eco-Explore CIC

[CR57] Vuarin P, Yves P (2014) Field evidence for a proximate role of food shortage in the regulation of hibernation and daily torpor : a review. J Comp Physiol B 184(6):683-97 10.1007/s00360-014-0833-0 10.1007/s00360-014-0833-0 24850375

[CR58] Webb PI, Skinner JD (1996) Summer torpor in African woodland dormice *Graphiurus murinus* (Myoxidae: Graphiurinae). J Comp Physiol B 166(5):325–330. 10.1007/BF02439919 8870263 10.1007/BF02439919

[CR59] Welsh Government. 2021. Ancient woodland inventory (Wales). Available at: https://datamap.gov.wales/layers/geonode:GWC21_Ancient_Woodland_Inventory_2021. Accessed 1 Jun 2022

[CR60] Wilsterman K, Ballinger MA, Williams CM (2021) A unifying, eco-physiological framework for animal dormancy. Funct Ecol 35(1):11–31. 10.1111/1365-2435.13718

[CR61] Wilz M, Heldmaier G (2000) Comparison of hibernation, estivation and daily torpor in the edible dormouse, *Glis glis*. J Comp Physiol B 170(7):511–521. 10.1007/s003600000129 11128441 10.1007/s003600000129

