# Supplementary material for: Environmental and population correlates of variation in short torpor use by wild hazel dormice (Muscardinus avellanarius)
Source: Oecologia. 2025 Jun 3;207(6):97. doi: 10.1007/s00442-025-05730-3 (PMC12134035; doi:10.1007/s00442-025-05730-3)
Supplement: Supplementary file 1 — Supplementary file1 (DOCX 27 KB) [file 442_2025_5730_MOESM1_ESM.docx]

**ELECTRONIC SUPPLEMENTARY INFORMATION 1.**

Summary of models testing relationships between hazel dormouse torpor and population parameters.

|  | **Measure** | **Model Type** | **Error Structure** | **Off-set** | **Years Tested** | **Age Classes Included** | **Additional information** |
| --- | --- | --- | --- | --- | --- | --- | --- |
| **1**. | Counts of adult dormice - whole season  (72,000 observations, 614 NDMP sites) | GLM | Poisson | Number of boxes checked in the survey season | Year T and Year T + 1 | Adults |  |
| **2**. | Counts of young dormice - whole season  (39,985 observations, 548 NDMP sites) | GLM | Poisson | Number of boxes checked in the survey season | Year T and Year T + 1 | Pinks, Grey-Eyes-Closed, Eyes Open, Juveniles |  |
| **3**. | Counts of adult dormice - early and late season  (72,000 observations 614 NDMP sites) | GLM | Poisson | Number of boxes checked in the survey season | Year T + 1 | Adults |  |
| **4**. | Counts of young dormice - early and late season  (39,985 observations, 548 NDMP sites) | GLM | Poisson | Number of boxes checked in the survey season | Year T and Year T + 1 | Pinks, Grey-Eyes-Closed, Eyes Open, Juveniles | Counts of young in Year T were tested using early and late torpor scores. Tests for Year T + 1 used the torpor score in Year T for the entire season. |
| **5**. | Breeding events  (28,082 surveys where breeding was monitored across 800 NDMP sites) | GLM | Binomial |  | Year T and Year T + 1 | Pinks, Grey-Eyes-Closed, Eyes Open | A breeding event was determined as any young age-class of dormice were found in a box the young are dependent on the mother therefore certain that the individuals were born during the current active season. |
| **6**. | Litter Size  (6,860 potential litters) | Mixed GLM using the package ‘lme4’ (Bates et al., 2015) | Poisson |  | Year T and Year T + 1 | Pinks, Grey-Eyes-Closed, Eyes Open | Mixed models were chosen to account for pseudo-replication within the model as yearly torpor score was repeated for each litter found within a site during any given survey year. |
| **7**. | Average mass of adult dormice (g)  (72,000 observations, 614 NDMP sites) | GLM | Gaussian |  | Year T and Year T + 1 | Adults (only respective sex in relation to Yearly Torpor Score). |  |
| **8.** | Average mass of Young Dormice (g)  (10,525 observations, 348 NDMP sites) | GLM | Gaussian |  | Year T and Year T + 1 | Eyes Open | Eyes Open was chosen as these are the most commonly encountered age class during surveys so allowed for the most data to be included into the subsequent models. |
